# Fast switching cholesteric liquid crystal optical beam deflector with polarization independence

**DOI:** 10.1038/s41598-017-06944-z

**Published:** 2017-07-26

**Authors:** Xiaobing Shang, Laurens Meeus, Dieter Cuypers, Herbert De Smet

**Affiliations:** 1 Ghent University and imec, Department of Electronics and Information Systems, Ghent, 9052 Belgium; 2 Ghent University, Department of Telecommunications and Information Processing, Ghent, 9000 Belgium

## Abstract

Optical beam deflectors based on the combination of cholesteric liquid crystals and polymer micro gratings are reported. Dual frequency cholesteric liquid crystal (DFCh-LC) is adopted to accelerate the switching from the homeotropic state back to the planar state. Polarization independent beam steering components are realized whose transmission versus the polarizing angle only varies 4.4% and 2.6% for the planar state and the homeotropic state, respectively. A response time of 451 ms is achieved for DFCh-LC-grating beam deflectors, which is fast compared to other nematic LC beam steerers with similar LC thickness.

## Introduction

Nowadays, more and more tunable beam steering or deflecting components are employed in newly emerging photonic and optical products, such as revolving mirrors for lighting or scanners^1^, adaptive concentrators for solar cell panels^2^, zoom lenses for digital cameras^3^, highly directional backlight systems for autostereoscopic displays^4, 5^ and so forth. Those applications are based on either electro-mechanical movements or micro-electro-mechanical systems (MEMS). Beam deflectors based on electromotors are usually bulky because of the macroscopic mechanical motions involved. Beam steering with MEMS is still difficult to implement for large beam sizes and low cost products.

Another available implementation of non-mechanical beam deflection is based on liquid crystal (LC). LC material usually has a large and field-tunable (electric field or magnetic field) birefringence, which is of great benefit to many novel optical applications. Beam steering components based on LCs are mainly categorized into two technical schemes:

- The first one has an uniform LC layer thickness (“cell gap”) between the two confining transparent substrates, but a location-dependent refractive index induced by spatially aligned liquid crystal directors. Normally a saw tooth like distribution of the optical path difference (OPD) is achieved by applying different voltage signals on the addressing electrodes^6^. One drawback of this type of device is that the deflection efficiency gets lower as the steering angle increases, largely due to the invalid flattened area on the phase profile caused by the intrinsic fringe field between two adjacent grating units^7, 8^. It is reported that the deflection efficiency of the LC grating with a large birefringence of 0.35 is about 56.5% as the steering angle is 5°, while further increasing the steering angle to 10°, results in an efficiency drop to 25%^9^. Another disadvantage is that accurate control of the phase profile within each unit is rather difficult for LC gratings with multiple addressing electrodes. One solution is to increase the number of electrodes and decrease the electrode width in each period, which unavoidably results in more complicated driving circuits and processing difficulty. To solve these issues, highly resistive or highly dielectric layers are adopted as an alternative method to smoothen the electric field, leading to more accurate OPD profiles and simpler driving circuits^10, 11^.
- The other LC beam steering technique utilizes a specifically designed topography on at least one of the confining transparent substrates, i.e. the device has a non-uniform cell gap. Since the LC material and the transparent substrate material are, two media with different refractive indices, the topography of the interface between these materials can be designed to achieve a certain optical function. Linear micro prism structures made on polymer substrates have been combined with well aligned nematic liquid crystals to achieve effective beam deflection^12, 13^. The advantage is that one can implement the desired beam steering performance more precisely and easily with the help of the specially designed micro optical structures^13, 14^. Its disadvantage is also caused by those micro optical structures, which result in higher operation voltages because the transparent conductive layer is usually positioned below the micro structure array, resulting in a large voltage drop over the micro structure material. Putting the conductive layers on top of the micro structures can reduce the operating voltage to some extent^15^, but this approach is difficult to implement and still needs higher driving voltages than the aforementioned LC grating with a gradient refractive index.

It is seen clearly what are the merits and demerits of each technique for LC beam steering technologies. Moreover, both LC beam steering techniques have a common issue – polarization dependence, which can be a severe problem for certain applications, such as efficient high flux lighting, smart glasses and smart contact lenses. LC beam deflectors with a polarizer reduce the light intensity by more than 50%, further hindering the applications of these LC beam steering technologies.

In this work, we try to improve the polarization independence of the LC beam steering devices based on the combination of liquid crystals and micro optical structures. Cholesteric liquid crystals are adopted to get less polarization dependent beam deflection for both the planar state and the homeotropic state. The electro-optical property and the response behaviour of the cholesteric LC beam steering components are also measured and characterized. This kind of beam steering devices is applicable for directional high-flux lighting, auto-focus contact lenses, highly efficient sunlight collectors and so forth.

### Principle

Unlike nematic liquid crystals (NLCs), cholesteric liquid crystals (Ch-LCs) exhibit a helical structure, whereby if one describes a trajectory along the helical axis, the orientation of the LC molecules encountered rotates by a full 360° around that axis over a period *P*_0_, also designated as the helical pitch. Normally the helical structures are induced either by the effect of a chiral dopant added to the NLCs or by the inherent molecular structure of some compounds such as cholesteryl benzoate^16^. If the helical axis of each domain is perpendicular to the confining substrates, the Ch-LC exhibits the so-called planar (P) state; Upon application of a high voltage difference between the transparent electrodes on both substrates, a strong electric field is established and the LC molecules try to reorient themselves parallel to this field, perpendicular to the substrates. As a result, the helical structures are unwound and the LC is now entering the homeotropic (H) state. In between the two states, there is another state, called the focal conic (FC) state, whereby the helical domains are randomly oriented. The P state strongly reflects light of wavelengths for which the following condition is satisfied:

1 $${\lambda }_{c}={n}_{ave}\times {P}_{0}$$

where *n*_*ave*_ = (*n*_*o*_ + *n*_*e*_)/2 being the average refractive index, a property which is employed frequently for the realization of reflective color displays^17–19^. For the proposed polarization independent beam steering devices, the central reflective wavelength *λ*_*c*_ can be set in either the ultra violet (UV) or the infrared (IR) ranges, thus the visible wavelengths would pass unhindered through the Ch-LC layers and experience the average refractive index of the LC material. By combining the Ch-LC with the micro optical structures, the incident light experiences a transition between different refractive indices at the LC-grating interface, leading to an unpolarized beam deflection. For the H state, the LC molecules are aligned vertically to the substrates so that an incoming perpendicular light beam always experiences the ordinary refractive index *n*_*o*_, regardless of its polarization direction. As a result, also in the H state polarization independent beam deflection is achieved.

## Results and Discussion

Based on the above idea and working mechanism, Ch-LC materials are combined with polymer micro grating substrates to assemble the polarization independent beam steering devices. The experimental results are described and discussed in detail for this section.

### Cholesteric LC beam steerer using E7 host LC

Since the operating voltage of Ch-LCs is inherently higher than that of the NLCs^20^, to reduce the driving voltage of the Ch-LC beam deflector, the conductive layer is deposited on top of the micro grating structure instead of underneath. A nematic LC-E7 (from Merck) with a large dielectric anisotropy (Δ*ε* ≈ 14) is adopted for the target Ch-LC material in order to further decrease the operation voltage. Its ordinary and extraordinary refractive indices at *λ* = 589 nm are *n*_*o*_ = 1.5224 and *n*_*e*_ = 1.7394, respectively. To lower the critical field unwinding the helical structure of the Ch-LC mixture^20^, and obtain high transmission of visible wavelengths, the Bragg reflective peak wavelength of the Ch-LC is set at *λ*_*c*_ = 2000 nm. This value is achieved with a concentration of 0.5 *wt*.% of the chiral dopant R5011 (from HCCH).

**Figure 1:**
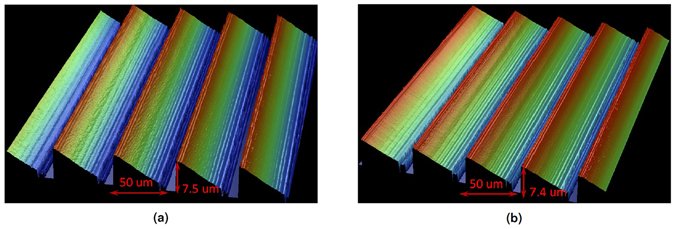
3D profiles of optical micro grating structures measured by using Wyko NT3300 profilometer: (**a**) Master grating on Epocore by laser ablation; (**b**) Replicated grating structure on NOA 74 UV cured resin layers by soft embossing.

The optical micro structures are fabricated using a technology based on laser ablation and soft embossing, as reported before^21, 22^. The pitch and the height of the UV grating structure are experimentally found to be 50 *μ*m and 7.4 *μ*m, respectively, which closely approximate those of the master Epocore grating, as shown in Fig. 1. A good planar state is expected to be beneficial for a good polarization independent beam steering performance, and the LC alignment layers are of vital importance in achieving and stabilizing different textures of Ch-LCs. Therefore, different LC alignment schemes are applied to evaluate the aligning effect on the planar state and the homeotropic state of the Ch-LC beam steerer. Since the conductive PEDOT layer becomes inactive (non-conducting) once it meets the polyimide (PI) solution, it is difficult to apply PI alignment layers on top of the PEDOT coated grating substrates. However, it is found that bare rubbing of the gratings also has the effect of aligning the LC molecules^13^. For that reason we have also utilized the bare rubbing procedure in our study. Four different LC aligning schemes are tried:

Scheme 1- bare grating rubbed along the grooves combined with flat indium tin oxide (ITO) counterpart coated with a rubbed PI layer to form anti-parallel alignment;

Scheme 2- bare grating rubbed along the grooves combined with flat ITO counterpart coated with a rubbed PI layer to form orthogonal alignment;

Scheme 3- bare grating without rubbing combined with flat ITO counterpart coated with a non-rubbed PI layer;

Scheme 4- bare grating without rubbing combined with bare flat ITO counterpart without any PI layers.

The total thickness of the LC layer of the samples is about 13 *μ*m. After the fabrication of empty LC cells with the four different alignment schemes, the Ch-LC mixture with the Bragg peak reflection in the IR range (*λ*_*c*_ = 2000 nm), which consists of the E7 host nematic LC and the chiral dopant R5011, is filled into the LC devices by vacuum filling.

**Figure 2:**
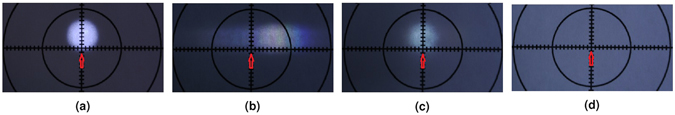
Collimated white light beam steering of Ch-LC-grating devices with E7 NLC and R5011 chiral dopant: (**a**) Original beam spot; (**b**) Beam deflection of Ch-LC steerer with *V*_*app*_ = 0; (**c**) Beam deflection of Ch-LC steerer with *V*_*app*_ = 200 *V*_*pp*_; (**d**) Beam scattering of Ch-LC steerer when the high voltage is removed abruptly.

The resulting beam steering performance with a collimated white light beam is shown in Fig. 2. Figure 2(a) depicts the original beam spot, which is projected onto a semi-transparent screen without passing through any samples. When the Ch-LC beam steering devices are inserted in the optical setup, due to the presence of the P state, the incident light rays are deflected to the right side of the original beam position, as shown in Fig. 2(b). The ultimate beam steering angle is determined by the following equations^23^:

2a $${n}_{p}\,\sin ({\theta }_{M}+{\theta }_{B})={n}_{LC}\,\sin \,{\theta }_{B}$$

2b $$\sin \,{\theta }_{st}={n}_{p}\,\sin \,{\theta }_{M}$$

where *n*_*p*_ and *n*_*LC*_ are the effective refractive indices for the polymer micro structure and the LC material, respectively; *θ*_*B*_ is the blaze angle of the micro grating (~10°); *θ*_*M*_ and *θ*_*st*_ are the angular position of the diffraction order with the maximum intensity and the ultimate beam steering angle of the LC component in the air, respectively. Using Eqs 2a and 2b, the ultimate beam steering angle *θ*_*st*_ is calculated to be 1.12°, which approximates the measured deflection angle 1.38° in the P state. Upon application of a high square wave voltage of 200 *V*_*pp*_, the LC textures transform into the H-state, and the beam spot shifts back to the original position due to the negligible refractive index difference between the LC and the polymer micro structure (Fig. 2(c)). After removing the applied voltage abruptly, the H state is changed into the FC state rather than the P state. The FC state scatters the incident light beam strongly due to the random orientation of the Ch-LC helical domains (Fig. 2(d)). This behaviour is not desired for optically transmissive beam steering components. If both substrates were flat as in traditional Ch-LC devices, the Ch-LC would transform into the P state immediately from the unwound H state by the rapid removal of the applied high voltage^24–26^, but this does not happen in the Ch-LC beam steering devices with any of the four different alignment schemes. It was observed that one hour after the removal of the high voltage from the H state, the FC state started to transform into the P state. A possible and plausible explanation is that the micro grating structures on one substrate play a role in retaining the thermodynamically metastable FC state, decelerating the transformation process to the stabler P state. To realize the desired behaviour, we need a solution to accelerate the recovery of the P state from the H state to achieve both the steering and the non-steering functionality for Ch-LC-grating beam deflecting devices.

### Dual frequency cholesteric LC beam steerer

**Figure 3:**
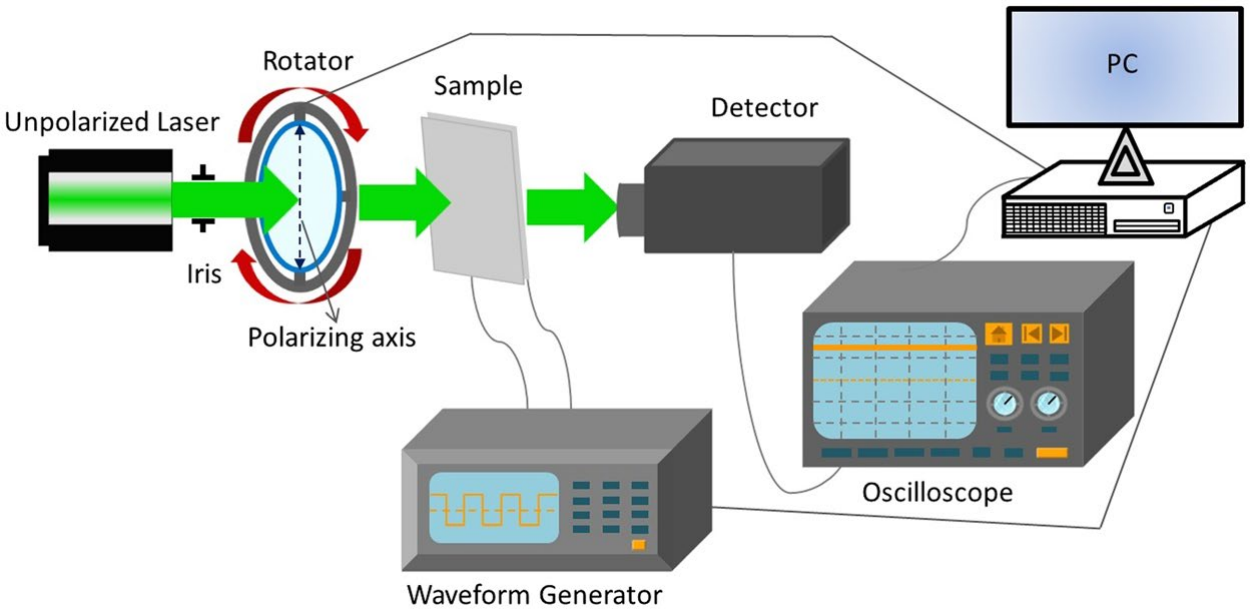
Schematic setup for optical measurements of Ch-LC-grating devices.

**Figure 4:**
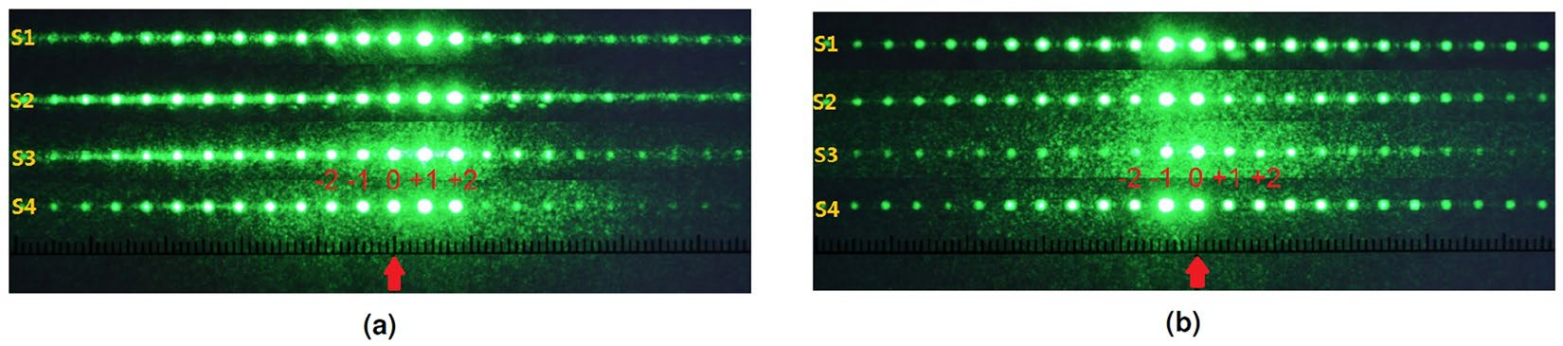
Green laser beam deflection by DFCh-LC-grating beam steering devices with (**a**) the P state and (**b**) the H state.

To accelerate the transformation from the H or FC texture to the P texture, dual frequency (DF) NLC-HEF951800-100 (from HCCH) is adopted as the host component and mixed with the same chiral dopant R5011 to form a DFCh-LC with its Bragg peak reflection also at *λ*_*c*_ = 2000 nm. The DFCh-LC is then filled into the devices with the aforementioned four different alignment schemes and the total LC thickness is about 13 *μ*m. An unpolarized He-Ne laser beam (*λ* = 543.5 nm) with a polarizer is used to characterize the beam steering performance, and the optical setup is depicted in Fig. 3. The laser beam steering patterns are demonstrated in Fig. 4. From the figures, it is seen that all the four samples exhibit similar beam deflecting behaviour in either the P state or the H state. When the laser beam is normally incident on the LC device having the P state, due to the refractive index difference between the DFCh-LC and the grating, the maximum intensity appears on the +1_*st*_ order, while if the device is in the H state due to the application of a voltage of 150 *V*_*pp*_, the maximum intensity is found to be at the −1_*st*_ order. Using the first order approximation angle derivation method^13^, the beam steering angles are obtained to be 0.61° and −0.24° respectively for the P state and the H state of the DFCh-LC-grating beam steering devices.

To verify the polarization independence of the steering characteristics, the transmission curve of the DFCh-LC beam steerers is measured as a function of polarizing angle of the incident light.

### The curves of transmission versus polarizing angle

**Figure 5:**
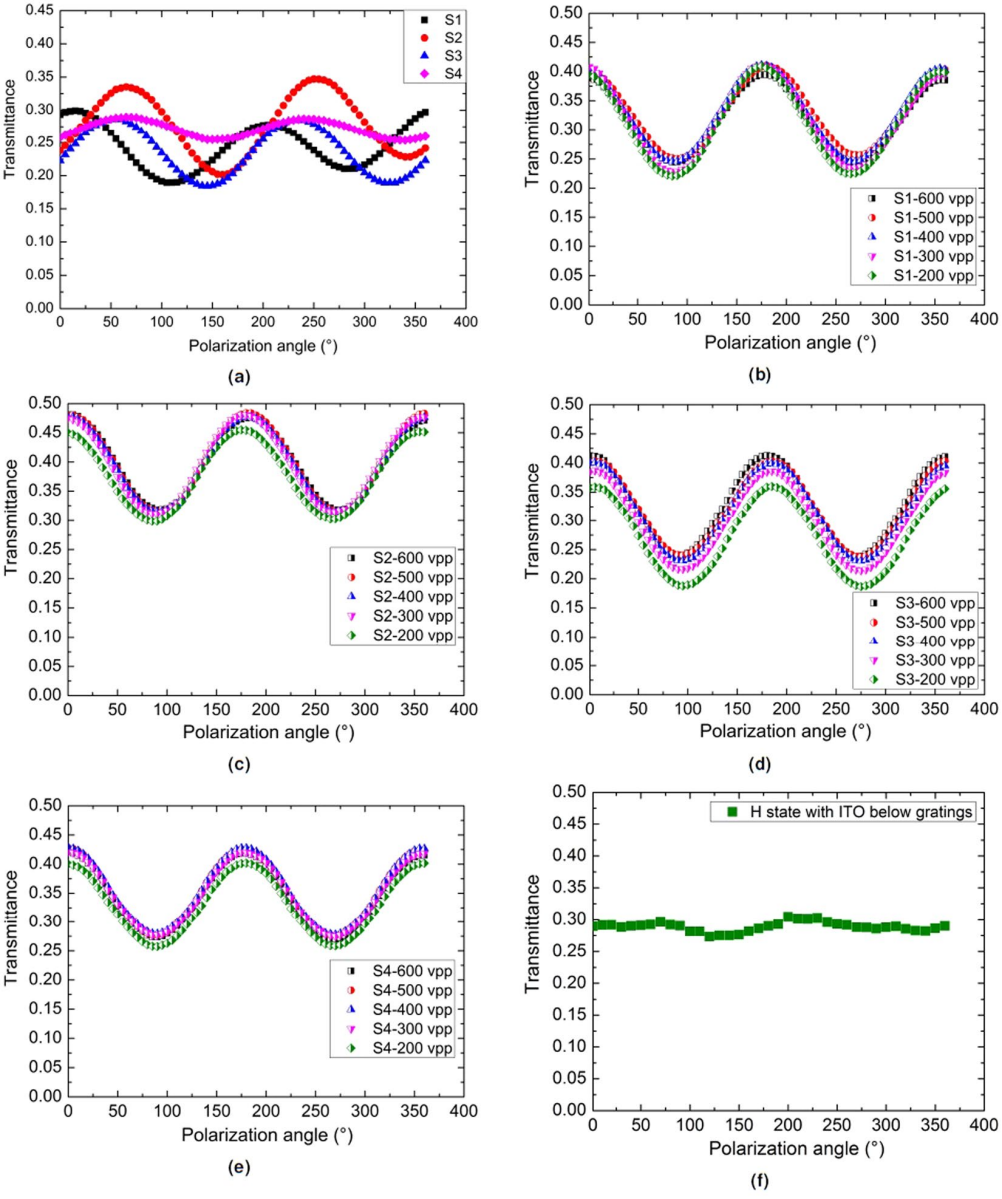
Transmission versus polarizing angle of the DF-Ch-LC beam deflectors in different states: (**a**) different schemes in P states; (**b**) H states of Scheme 1; (**c**) H states of Scheme 2; (**d**) H states of Scheme 3; (**e**) H states of Scheme 4; (**f**) H state of DF-Ch-LC grating devices with the ITO layer below micro gratings by applying *V*_*app*_ = 600 *V*_*pp*_.

Figure 5(a) shows the measurement result for the components with four different LC alignment schemes in their P state.

It is found that Scheme 4 has the lowest transmission variation (4.4%), while Scheme 2 has the largest transmission variation (16.4%); Scheme 1 and Scheme 3 have intermediate variation values (13.8% and 15% respectively). This can be explained by the fact that planar alignment layers exert a positive anchoring effect to favor the P texture, which causes a preferential orientation of the LC molecules. Especially near the substrate a fraction of the LC molecules is aligned strongly along the PI rubbing direction, resulting in the observed polarization dependency of the transmittance curves. Figure 5(b)–(e) show the curves of the transmission versus the polarizing angle for beam steering devices in the H state, also for the four different LC alignment schemes. It is observed that with *V*_*app*_ = 200 *V*_*pp*_, the transmission variation values of the H state (S1-20.7%, S2-14.6%, S3-22.6% and S4-15.6%) are higher than those of the aforementioned P state. Even when applying much higher voltages (up to 600 *V*_*pp*_), the polarization dependency does not seem to decrease at all. With strong electric fields, the helical structures of DFCh-LCs are unwound, and most of the LC molecules reorient parallel to the electric field. If the electric field is perpendicular to the substrates, the incident light beam experiences the same refractive index (the ordinary index, *n*_*o*_ = 1.496) of the DFCh-LCs regardless of polarization direction, no or negligible polarization dependent transmission variation is expected. In our case however, the conductive PEDOT:PSS layer is deposited on top of the gratings in order to reduce the operation voltage of the beam steering components. The conductive layer on the tilted slopes in combination with the counterpart full electrode on the other glass substrate generates slanted electric fields, which realign the LC molecules along a direction that deviates from the normal direction. When increasing the applied voltage, the inclined electric fields become stronger, but this does not cause more LC molecules to be aligned perpendicular to the substrates. The residual birefringence caused by the reoriented LC molecules leads to a certain polarization dependency of the transmission curves. That is the reason why the transmission variation of the H states is almost unchanged even with higher applied voltages. It is expected that with the conductive layer below the grating structures, the slanted electric field could be relieved to some extent due to the electric field redistribution caused by the dielectric polymer layers, thus improving the polarization independence of the H state for Ch-LC beam steering components. We have indeed verified this experimentally and Fig. 5(f) shows the polarization dependent transmission curve of the H state by applying *V*_*app*_ = 600 *V*_*pp*_, for the DF-Ch-LC grating devices based on Scheme 4 but with the ITO layer below the micro grating structures. It is seen that the transmission variation (2.6%) is much lower than that of the H states for the DFCh-LC-grating beam steering components with the conductive layer on top of the gratings, which is even smaller than the best P state of Scheme 4. Hither, it can be concluded that the beam steering component with the combination of DFCh-LCs and polymer gratings based on Scheme 4, i.e. the one without any LC alignment, has the best polarization independence among the four different LC alignment methods.

### The frequency dependent transmission curve

**Figure 6:**
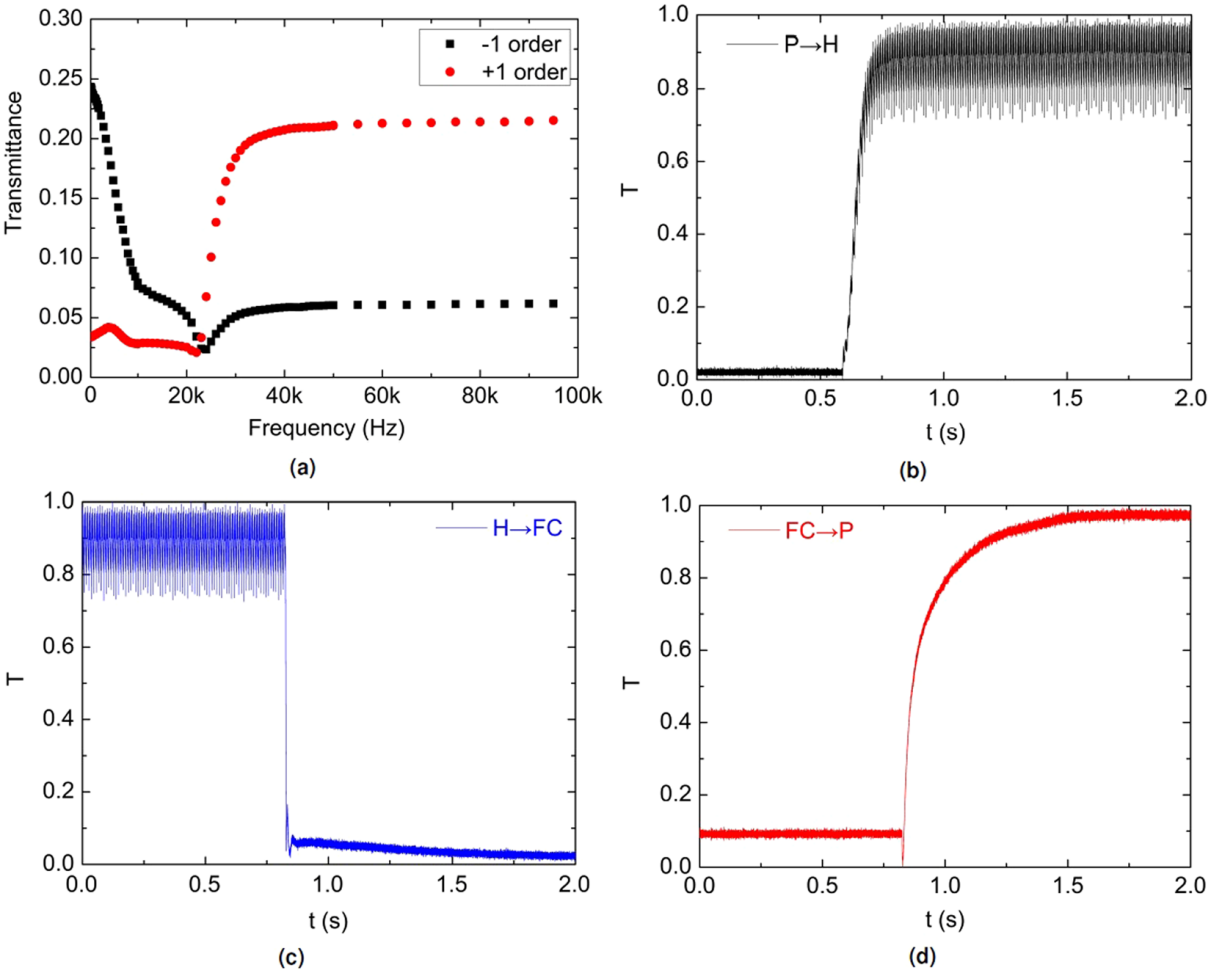
The switching behaviour of DFCh-LC-grating beam steering devices between different states: (**a**) transmittance versus applied frequency; (**b**) switching from P to H; (**c**) switching from H to FC; (**d**) switching from FC to P.

In order to get the optimized condition for the dual frequency driving, the transmission versus applied frequency curve of the DFCh-LC-grating beam steering device based on Scheme 4 has been characterized, the results are shown in Fig. 6(a). The measurements start with the P state, which is realized by applying *V*_*app*_ = 200 *V*_*pp*_ and f = 50 kHz. The detector is first put at the +1_*st*_ order where the maximum intensity is located at the presence of the P state, then a high voltage of 150 *V*_*pp*_ starting from lower frequencies to higher ones is applied to the device. The same procedures are also applied to the −1_*st*_ order where the maximum intensity of the H state resides. By applying strong electric fields with lower frequencies, the DFCh-LC exhibits a positive dielectric anisotropy (Δ*ε* = +2.6 at f = 1 kHz). The net torque exerted by the two electric field components which are respectively parallel and perpendicular to the long LC molecule axis, results in the LC directors reorienting along the direction of the electric field. The helical structure is unwound and the device enters the H state. The maximum intensity shifts to the −1_*st*_ order (referring to the black squares), the intensity at the +1_*st*_ order is therefore significantly decreased (referring to the red dots). When increasing the applied frequency, the positive dielectric anisotropy starts decreasing. Hence, the net torque for realigning the LCs into the H state becomes weaker and some scattering FC domains appear among the dominant H textures. The scattering FC textures result in a direct reduction of the transmittance at the −1_*st*_ order (as seen in the curve with black squares), while the transmittance at the +1_*st*_ order stays relatively stable. At f = 23 kHz, the transmittance of the +1_*st*_ order starts to rise, while the intensity of the −1_*st*_ order reaches the bottom of its curve. This phenomenon indicates that the dielectric anisotropy of the DFCh-LC has been transformed into a negative value and the LC directors try to reorient perpendicularly to the electric field, which contributes to the formation of the P texture. When the frequency is further increased, the absolute value of the negative dielectric anisotropy becomes larger (Δ*ε* = −3.2 at f = 100 kHz) and the resultant net torque is further enhanced, leading to more P texture and a higher transmittance at the +1_*st*_ order. Above f = 50 kHz, the DFCh-LCs are completely transformed into the P texture, and both transmission curves are saturated.

### The response behaviour of DF-Ch-LC beam deflector

Based on the above measurements, it is seen that the applied frequency above 50 kHz leads to a constant transmission. Hence, the driving frequency is fixed at *f*_*H*_ = 50 kHz and *f*_*L*_ = 100 Hz with driving voltage of *V*_*app*_ = 160 *V*_*pp*_. The response time of the DFCh-LC–grating beam steering components are characterized between different states, the results are illustrated in Fig. 6(b)–(d). The switching time is defined as the period during which the transmittance is changed from 10% (90%) to 90% (10%). The response time from P to H is measured to be 112 ms (Fig. 6(b)). Upon removal of the applied voltage, the switching time from the H state to the FC state is found to be 15 ms (Fig. 6(c)). The switching time from FC to P is 324 ms (Fig. 6(d)). The total switching time of the DFCh-LC-grating beam deflector is 451 ms, which is much quicker than the NLC based beam steering devices with several seconds of switching time in a similar LC thickness^10–13^.

## Conclusion

Cholesteric liquid crystal was combined with micro grating structures to build polarization independent beam steering devices. Dual frequency cholesteric liquid crystal is adopted to accelerate the switching from the homeotropic state back to the initial planar state. The polarization dependence of the transmission is as low as 4.4% for the P state and 2.6% for the H state if the conductive layer is positioned below the grating structures. The observed response time for a 13 *μ*m thick LC layer (~451 ms) is much lower than that of NLC beam steering devices with similar thickness. This kind of polarization independent beam steering components could be used in many novel optical applications such as highly efficient solar cell concentrators, micro beam deflector arrays for autostereoscopic displays, polarization independent auto-focus contact lenses and so forth.

## Methods

### Device fabrication

A KrF nano-second laser beam (*λ*=248 nm) combined with a shadow mask with a trapezoidal aperture is used to scan the Epocore (from micro resist technology GmbH) layer on glass substrates. The optimized laser processing parameters are F = 400 mJ/cm^2^, f = 100 Hz and v = 0.25 mm/s. To get the desired refractive index of the micro gratings, another replication process with polydimethylsiloxane (PDMS) molds and UV curable resins was developed. With the negative PDMS mold derived from the laser ablated Epocore master grating, micro optical grating structures are replicated in an UV curable NOA 74 (from Norland Products Inc., *n*_*p*_ = 1.52) resin layer on the glass substrates. The UV irradiation for NOA 74 is *I* = 60 *mW*/*cm* ^2^, *λ* = 365 nm for 5 minutes.

PEDOT:PSS (from Sigma-Aldrich) with a sheet resistance *R*_□_ ≤ 100 Ω/□ is adopted for the formation of the transparent conductive layers. In order to improve the surface wetting so that good conductive layers are obtained on top of the grating structures, a low pressure plasma surface treatment is first applied to the grating substrate using a PICO reactor (from Diener electronic GmbH + Co. KG), with 0.8 mbar air pressure and a power of 100 W (50% of full power) for 1 minute. The PEDOT:PSS is subsequently spin coated at 1000 rpm for 35 seconds. The grating substrates with the PEDOT layer are then baked on a hotplate at 90 °C for 10 minutes. To avoid short circuits between the PEDOT coated grating substrate and the flat indium tin oxide (ITO) counterpart, a thin isolating NOA 74 resin layer (1.30 ± 0.05 *μ*m in thickness) is spin coated on the ITO layer and UV cured. A polyimide layer is subsequently spin coated on the flat ITO glass substrate and thermally cured. The grating substrate and the ITO counterpart are assembled to form empty LC cells, which are filled with liquid crystals by vacuum filling.

### Optical characterization

The unpolarized laser beam with *λ* = 543.5 nm (Model 1652, JDS Uniphase) is first passing through a polarizer, which is installed onto a rotational stage. The linearly polarized light beam is incident onto the sample and deflected by the device, which is driven by an arbitrary waveform generator with applying the required voltage signals. The steered light is collected by a detector whose data are read by an oscilloscope. With an updated polarizing direction of the polarizer, the above process iterates. The rotator, the waveform generator and the oscilloscope are all controlled by a computer through our designed Python program for the measurement of polarization independence.

### Data Availability

The datasets generated during and/or analysed during the current study are available from the corresponding author on reasonable request.
